# Topical administration of tacrolimus and corticosteroids in tapering doses is effective in preventing immune rejection in high-risk keratoplasty: a 5-year follow-up study

**DOI:** 10.1186/s12886-022-02318-w

**Published:** 2022-03-04

**Authors:** Xiaolin Qi, Lichao Wang, Xiaoyu Zhang, Min Liu, Hua Gao

**Affiliations:** 1 State Key Laboratory Cultivation Base, Shandong Provincial Key Laboratory of Ophthalmology, Eye Hospital of Shandong First Medical University, Shandong Eye Institute, Shandong First Medical University & Shandong Academy of Medical Sciences, Jinan, China; 2 Shandong Eye Hospital, 372 Jingsi Road, 250021 Jinan, China

**Keywords:** Keratoplasty, Immune rejection, Tacrolimus, Cyclosporine, Corticosteroids

## Abstract

**Background:**

To evaluate the efficacy of the topical administration of immunosuppressants and corticosteroids in tapering doses in the management of patients with high-risk keratoplasty.

**Methods:**

One hundred and six patients treated with topical immunosuppressants (50 eyes in the FK506 group and 56 eyes in the CsA group) and corticosteroid eye drops in tapering doses were enrolled in the study. The rates of rejection episodes, irreversible rejection, graft survival, and related influential factors were evaluated.

**Results:**

The mean follow-up period was 48.1 ± 7.9 months (range, 36–60 months). The rates of rejection episodes and irreversible rejection were 14.0% and 6.00% in the FK506 group and 37.5% and 7.1% in the CsA group, respectively. Kaplan-Meier survival analysis demonstrated a significantly higher graft survival rate in the FK506 group (81.6%±5.3%, 71.1%±6.3%) compared with that in the CsA group (71.1%±6.3%, 57.5%±7.5%) at 3 and 5 years after surgery (*P* = 0.006). Multivariate logistic regression revealed that preoperative risk score ≥ 3 (*P* = 0.016) and endothelial immune rejection (*P* = 0.033) were risk factors associated with graft survival.

**Conclusions:**

Topical administration of tacrolimus and corticosteroids in tapering doses is effective in decreasing the incidence of immune rejection in high-risk keratoplasty. Careful instruction of patients on the reasonable use of topical tacrolimus is critical to avoid immune rejection induced by sudden discontinuation of medication.

## Background

High-risk keratoplasty is defined as having at least two quadrants of stromal vascularization and/or a history of previous graft rejection. Other risk factors include chemical burns, corneal graft diameters exceeding 9 mm, perforation or ocular inflammation at the time of surgery, and a low recipient age [1–4]. Immune rejection in high-risk keratoplasty remains a therapeutic challenge for eye doctors. Doctors have made many efforts to examine the variety and usage of anti-rejection medications, [5–8] but there is still a lack of an ideal treatment strategy.

Corticosteroid therapy remains the mainstay method of preventing corneal graft rejection because of its dramatic inhibition of dendritic cell (DC) differentiation and maturation and restoration of a noninflamed microenvironment to support the transplanted graft [9]. However, the regimen used among respondents in the Cornea Society survey varied widely. Long-term use of corticosteroid eye drops is not recommended due to underlying side events, such as increased intraocular pressure or cataracts.

Topical cyclosporine (CsA) has been prescribed for years to treat different immune diseases of the eye. However, the majority of prospective studies have failed to demonstrate any benefit from the use of topical CsA for high-risk keratoplasty [5, 7, 10]. Tacrolimus (also named FK506) has been extensively used in preventing immune rejection for human organ transplantation. However, few case series have reported the beneficial effects of topical tacrolimus in human high-risk corneal transplantation [1, 6, 7, 11]. In this study, we aimed to evaluate the efficacy of topical tacrolimus and CsA during 5 years of follow-up and concluded that topical administration of tacrolimus and corticosteroid eye drops in tapering doses is effective in preventing immune rejection in high-risk keratoplasty.

## Methods

### Patients

This study was approved by the Institutional Review Board of Eye Hospital of Shandong First Medical University and adhered to the tenets of the Declaration of Helsinki. When the study was conducted, the National Medical Products Administration has not approved the use of topical eye drops containing 0.1% tacrolimus (Senju Pharmaceutical Ltd) in the treatment of immune rejection. Therefore, before participation in the study, the potential benefits and theoretical risks of the off-label use of topical tacrolimus were fully described and explained, and written informed consent was obtained from all the involved patients.

One hundred six consecutive patients (106 eyes) undergoing high-risk keratoplasty at Eye Hospital of Shandong First Medical University from Jan 2013 to Jan 2015 were included in the study. The preoperative risk score for all patients was recorded according to the method reported by Sloper CM et al. [2]. of which each chemical burn scored as 4, herpes simplex keratitis as 2, and previous graft rejection, grafts diameter ≥ 9 mm, infectious keratitis as 1, respectively (Table 1). The patients were divided into two groups in accordance with their desired treatment: the FK506 group was treated with 0.1% tacrolimus and corticosteroid eye drops, and the CsA group was treated with 1% CsA (North China Pharmaceutical Group Corporation, NCPC) and corticosteroid eye drops. Patients with untreated glaucoma, cataracts, or retinal detachment were not included.

**Table 1:** Preoperative characteristics of patients in FK506 group and CsA group

Characteristics	FK506 group	CsA group	*P*
**No. Eyes**	50	56	
**Age, y**			
Mean(SD)	51.2(11.9)	48.1(12.3)	0.521
Range	11–67	12–70	
**Sex**			
Male: Female	26:24	32:24	0.342
**Preoperative Risk Factors**			
** Previous graft rejection** Chemical burn Infectious keratitis	9 3 6	10 3 7	0.617
** Stromal vascularization ≥ two quadrants** Herpes simplex keratitis	9 9	9 9	1.000
** Chemical burn** Acid burn Alkali burn	7 3 4	7 3 4	1.000
** Grafts diameter ≥ 9 mm** Bullous keratopathy Corneal leucoma	14 7 7	16 8 8	0.562
** Infectious keratitis** Bacterial keratitis	11 11	14 14	0.430
**Surgical Treatment**			
Penetrating keratoplasty	45	51	0.571
Keratolimbal allograft	5	5	1.000

Immune rejection was classified as epithelial, stromal, and endothelial rejection by virtue of the following signs: an epithelial rejection line, subepithelial infiltrates, graft edema not previously noted, an endothelial rejection line, new keratic precipitates, and/or cells in the anterior chamber. [3, 12, 13].

### Postoperative therapy

**Fig. 1:**
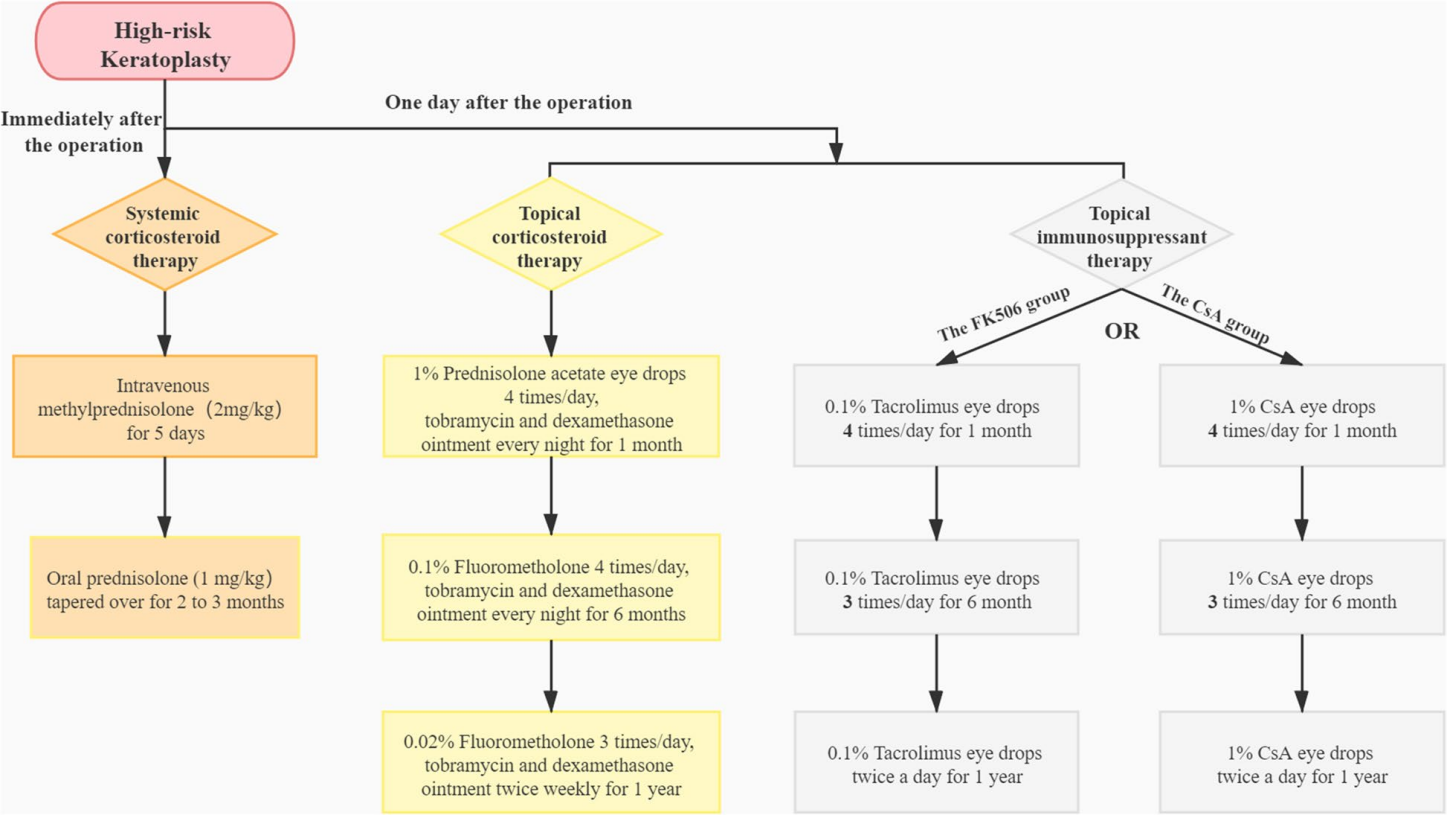
The flowchart of systemic and topical treatment strategy after high-risk keratoplasty

The treatment strategy used for systemic and topical corticosteroid therapy for all patients was consistent. During the first 5 days after the operation, all patients received intravenous methylprednisolone of 2 mg/kg/day. Then, the patients were treated with oral prednisolone, with an initial dose of 1 mg/kg/day and the dose was gradually tapered over a period of 2 to 3 months. In addition, topical 1% prednisolone acetate eye drops were prescribed 4 times a day for 1 month postoperatively. Subsequently, 0.1% fluorometholone was administered 4 times daily for 6 months and then, it was replaced by 0.02% fluorometholone, which was used three times a day for at least 1 year. The combination of tobramycin and dexamethasone ophthalmic ointment was applied every night for 6 months and then the frequency was tapered to twice weekly [13–16]. In addition, the patients were instructed to use 0.1% tacrolimus eye drops or 1% CsA eye drops 4 times a day for 1 month and then, the frequency was tapered to three times per day for 6 months and twice daily for at least 1 year (Fig. 1). For patients with dry eye disease, 0.3% sodium hyaluronate eye drops or carbomer gel was prescribed according to patient conditions.

### Antirejection therapy

The patients with transplant rejection received intravenous methylprednisolone of 2 mg/kg each day for 5 to 7 days, followed by oral prednisolone of 1 mg/kg daily and the dose was gradually tapered over a period of 1 to 2 months. For the first 3 days, tobramycin and dexamethasone eye drops were given every 2 h and then tapered to 4 times daily for the next 2 to 3 weeks. Then, 0.02% fluorometholone eyedrops were administered 4 times per day. In addition, tobramycin and dexamethasone ophthalmic ointment was given every night for 1 month and then tapered to twice each week [13–15]. At the same time, 0.1% tacrolimus eye drops or 1% CsA eye drops were used 4 times daily according to the patient’s condition.

### Main outcome measures

The patient history, demographic information, preoperative risk factors, onset time of immune rejection, symptoms, medication compliance, and days of interval (the time between discontinuation of the drugs and onset of immune rejection) were recorded. Best-corrected visual acuity (BCVA), intraocular pressure and slit-lamp examinations were performed.

### Statistical analyses

All data are described as the mean value ± standard deviation. Statistical analyses were performed using SPSS 22.0 (SPSS, Chicago, Illinois, USA). A *P* value of ≤ 0.05 was considered statistically significant. The demographics and preoperative risk score were compared with the Wilcoxon signed rank test between the two groups. Kaplan-Meier survival analysis and log rank tests were performed to evaluate graft survival. The most highly influential factors, including age, gender, preoperative risk score (≥ 3 or < 3), surgical treatment (penetrating keratoplasty or keratolimbal allograft), use of topical immunosuppressants (0.1% tacrolimus eye drops or 1% CsA eye drops), poor medication compliance when using 0.1% tacrolimus eye drops or 1% CsA eye drops, and type of immune rejection (endothelial or nonendothelial immune rejection) were analyzed using multivariate adjusted logistic regression.

## Results

The mean follow-up period was 48.1 ± 7.9 months (range, 36–60 months). Sixty-eight patients were male, and forty-eight patients were female. The mean age was 49.7 ± 12.2 years (range, 11–70 years). The demographics and preoperative risk scores of the two groups were comparable, and the intergroup comparisons showed no significant differences (Table 1).

### Rejection episodes

In the FK506 group, immune rejection was observed in 7 eyes, with stromal rejection in 3 eyes and endothelial rejection in 4 eyes, and the rate of rejection episodes was 14.0% (7/50). The causative factor was discontinuation of the FK506 eye drops in 7 eyes, as the other drugs were administered continuously (with an average interval of 4.9 ± 0.2 days). Rejection occurred within 6 months after surgery in 4 eyes, at 6 months to 1 year in 1 eye, at 1 year to 2 years in 1 eye, and at 2 years to 3 years in 1 eye. The graft in four eyes was restored to a clear graft after 7.9 ± 1.40 days of antirejection therapy. However, the corneal grafts were continuously edematous and opaque in 3 eyes. The rate of irreversible rejection was 6.00%.

In the CsA group, immune rejection was observed in 21 eyes, with stromal rejection in 4 eyes and endothelial rejection in 17 eyes. The rate of rejection episodes was 37.5% (21/56). The causative factors included poor compliance with all kinds of eye drops in 5 eyes, discontinuation of the use of corticosteroid eye drops in 6 eyes (with an average interval of 8.4 ± 2.3 days), and discontinuation of the use of CsA eye drops in 10 eyes (with an average interval of 6.3 ± 2.5 days). Rejection occurred within 6 months after surgery in 8 eyes, at 6 months to 1 year in 7 eyes, at 1 year to 2 years in 3 eyes, and at 2 years to 3 years in 3 eyes. The graft in seventeen eyes was restored to a clear graft after 8.8 ± 2.2 days of antirejection therapy. However, the corneal grafts were continuously edematous and opaque in 4 eyes. The rate of irreversible rejection was 7.1%.

### Graft survival

**Fig. 2:**
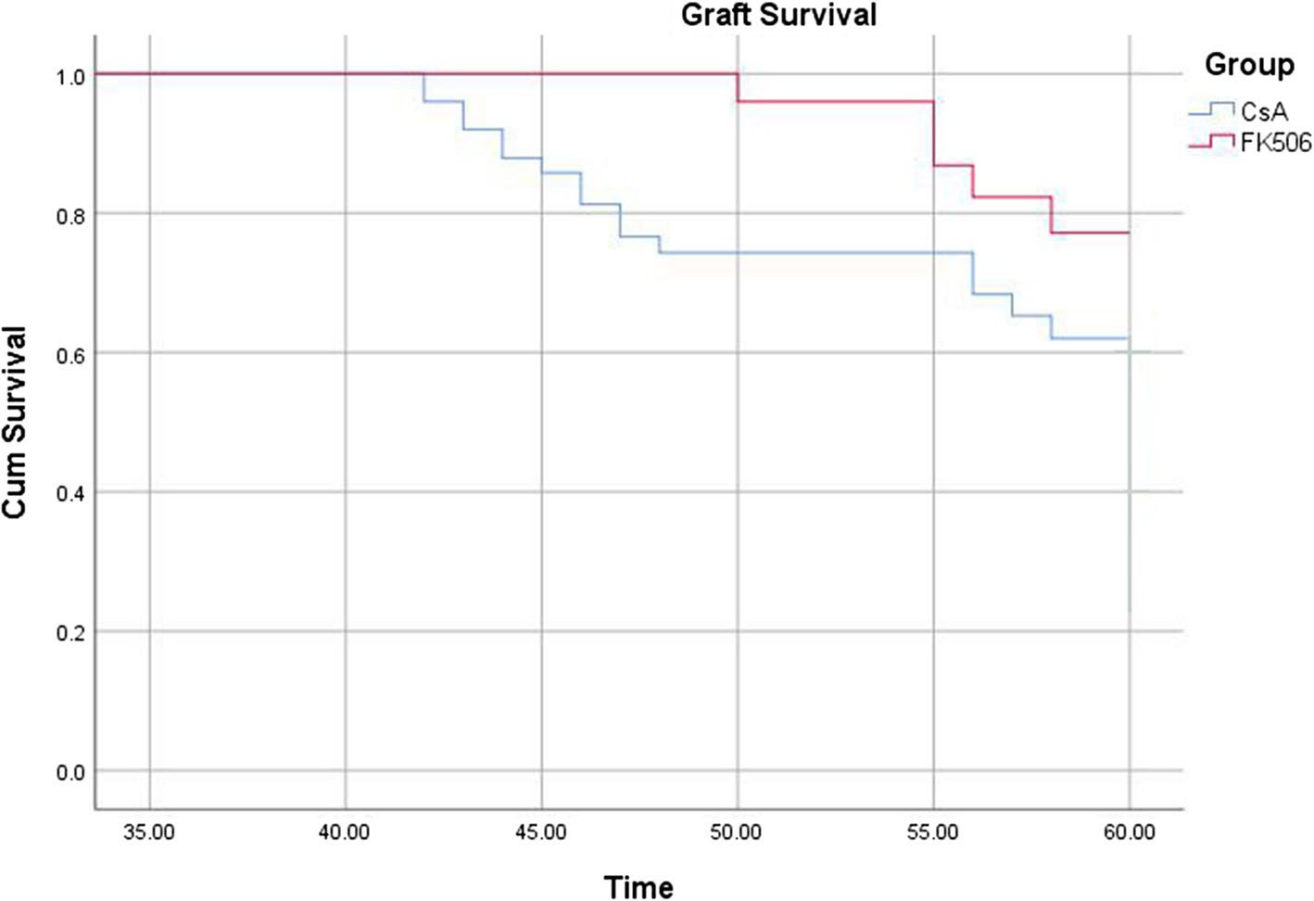
Kaplan-Meier curve of graft survival rate for patients who followed the treatment regimen of FK506 group and CsA group at 3 and 5 years after surgery

The graft survival rate was 81.0%±7.4% and 72.0%±8.9% at 3 years after surgery and 71.9%±6.2% and 61.2%±6.9% at 5 years after surgery in the FK506 group and CsA group, respectively. After patients with discontinuation of medication or poor medication compliance have been excluded, Kaplan-Meier analysis and log rank tests were performed for patients who followed the treatment regimen (43 eyes in the FK506 group, and 35 eyes in the CsA group), the results showed that patients in the FK506 group had a significantly higher graft survival rate at both 3 and 5 years after surgery than patients in the CsA group (Fig. 2), and the difference was statistically significant (*P *= 0.003).

### Influential factors

Graft survival was correlated with the preoperative risk score (≥ 3, *P* = 0.016) and endothelial immune rejection (*P* = 0.033) (Table 2).

**Table 2:** Influential factors for graft survival

Variable	No. Eyes	P value	RR(95% CI)
**Age**		0.746	1.208(0.385,3.786)
**Sex**	68 48	0.739	0.708(0.093,5.400)
Male			
Female			
**Preoperative Risk Score**	28 78	0.016	4.161(1.307,13.250)
≥ 3			
< 3			
**Surgical Treatment**	96 10	0.812	0.770(0.090,6.621)
Penetrating keratoplasty			
Keratolimbal allograft			
**Topical Immunosuppressants**	56 50	0.676	0.964(0.109,7.121)
1% CsA			
0.1% Tacrolimus			
**Poor Medication Compliance**	10 7	0.604	1.667(0.131,7.551)
1% CsA			
0.1% Tacrolimus			
**Type of Immune Rejection**	21 7	0.033	3.532(1.109,11.251)
Endothelial			
Non-endothelial			

### Side effects

The common side effects were redness (15 eyes), burning (14 eyes), and a stinging sensation (14 eyes) after drug instillation, which occurred more often in the CsA group (redness in 8 eyes, burning in 9 eyes, and stinging in 9 eyes) than in the FK506 group. No cataracts or elevation of intraocular pressure were detected in the two groups during the follow-up.

## Discussion

Immune rejection after corneal transplantation remains the leading cause of graft failure. The 5-year survival rates of corneal grafts are dramatically decreased for high-risk keratoplasty and range between 25% and 65% [17–21]. Since corticosteroids are insufficient in preventing graft rejection in high-risk patients, [22] topical administration of immunosuppressants such as FK506 and CsA is preferred. However, limited data exist on the efficacy of topical immunosuppressants in preventing immune rejection after high-risk keratoplasty [1, 6, 7, 11]. In our study, we applied 0.1% tacrolimus and corticosteroid eye drops in tapering doses to patients with high-risk keratoplasty and concluded that they are effective in reducing immune rejection and prolonging graft survival.

Corticosteroids are currently the mainstay of postoperative antirejection therapy. Given that the peak time of immune rejection was 1 to 3 months after high-risk keratoplasty, [13] intensive topical and intravenous steroids were administered within 1 month after surgery and tapered over a period of 2 months. Afterwards, fluorometholone was prescribed and tapered to a maintenance dose. Fluorometholone eye drops rapidly formed inactive metabolites in the corneal tissue, and only a small proportion passed through the cornea into the aqueous humor, [23] thus reducing the possibility of side effects associated with the elevation of intraocular pressure or cataracts. Unlike the results of Zhai et al., rejection was observed 30 months after surgery in 4 eyes in our study, resulting in irreparable loss of graft endothelial cells [24]. Therefore based on our experience, a maintenance dose of corticosteroid eye drops during the follow-up period is indicated for most patients, and regular detection of intraocular pressure is worthwhile.

Compared with CsA emulsion, tacrolimus is more hydrophilic with a higher transcorneal diffusion rate, making it a potential candidate for the treatment of corneal graft rejection [25]. Recent clinical studies have demonstrated that topical administration of tacrolimus was safe and effective in reducing the incidence of graft rejection and irreversible rejection [1, 6, 11, 26]. But few case series have reported its beneficial effects of prolonging graft survival rate, which was 81.0%±7.4% and 71.9%±6.2% at 3 and 5 years after surgery, significantly higher when compared with that conducted by Chow et al. [4] in addition, systemic side effects on blood pressure, renal function, and liver function were consequently avoided.

The efficacy of tacrolimus as an immunosuppressive agent is 10–100 times higher than that of CsA, [8] but sudden discontinuation of tacrolimus is more likely to induce immune rejection, with an average interval of 4.9 ± 0.2 days (shorter than that of CsA), which was not observed in the study of Zhai et al [24]. In addition, the restoration of corneal clarity after immune rejection is directly related to the interval from symptom onset to treatment and the degree of the immune response. The longer the interval is and/or the more severe the endothelial rejection is, the harder it will be to restore corneal transparency (*P* = 0.033).

The limitation of the study is the difference in medication compliance between the two groups (discontinuation in 21 eyes in the CsA group, far beyond the 7 eyes in the FK506 group), which would affect the results of the rejection episodes rate and graft survival. The reason for the large number of discontinuations in the CsA group was mainly due to the ocular surface discomfort. The side effects of redness, burning and a stinging sensation after drug instillation often appeared. Some patients were very sensitive to these side effects and they questioned the treatment plan, which resulted in poor medication compliance, and even self-discontinuation.

## Conclusions

In conclusion, topical administration of tacrolimus and corticosteroids in tapering doses is effective in decreasing the incidence of immune rejection and significantly prolonging graft survival in high-risk keratoplasty. It is critical to recommend the reasonable use of topical tacrolimus to patients, thus avoiding inducing immune rejection by sudden discontinuation of medication. Further randomized controlled trials are required to provide more objective evidence to evaluate medication efficacy.

## Data Availability

The datasets used and/or analysed during the current study are available from the corresponding author on reasonable request.

## Abbreviations

FK506: Tacrolimus
CsA: Cyclosporine
LK: lamellar keratoplasty
BCVA: Best-corrected visual acuity
DC: Dendritic cell
